# Bioaccessibility of Pb from Ammunition in Game Meat Is Affected by Cooking Treatment

**DOI:** 10.1371/journal.pone.0015892

**Published:** 2011-01-14

**Authors:** Rafael Mateo, Ana R. Baos, Dolors Vidal, Pablo R. Camarero, Monica Martinez-Haro, Mark A. Taggart

**Affiliations:** Instituto de Investigación en Recursos Cinegéticos (IREC), CSIC-UCLM-JCCM, Ciudad Real, Spain; University of Western Ontario, Canada

## Abstract

**Background:**

The presence of lead (Pb) ammunition residues in game meat has been widely documented, yet little information exists regarding the bioaccessibility of this Pb contamination. We study how cooking treatment (recipe) can affect Pb bioaccessibility in meat of animals hunted with Pb ammunition.

**Methodology/Principal Findings:**

We used an *in vitro* gastrointestinal simulation to study bioaccessibility. The simulation was applied to meat from red-legged partridge (*Alectoris rufa*) hunted with Pb shot pellets and cooked using various traditional Spanish game recipes involving wine or vinegar. Total Pb concentrations in the meat were higher in samples with visible Pb ammunition by X-ray (mean±SE: 3.29±1.12 µg/g w.w.) than in samples without this evidence (1.28±0.61 µg/g). The percentage of Pb that was bioaccessible within the simulated intestine phase was far higher in meat cooked with vinegar (6.75%) and wine (4.51%) than in uncooked meat (0.7%). Risk assessment simulations using our results transformed to bioavailability and the Integrated Exposure Uptake Biokinetic model (IEUBK; US EPA) show that the use of wine instead of vinegar in cooking recipes may reduce the percentage of children that would be expected to have >10 µg/dl of Pb in blood from 2.08% to 0.26% when game meat represents 50% of the meat in diet.

**Conclusions/Significance:**

Lead from ammunition in game meat is more bioaccessible after cooking, especially when using highly acidic recipes. These results are important because existing theoretical models regarding Pb uptake and subsequent risk in humans should take such factors into account.

## Introduction

Small fragments of Pb can be deposited along Pb shot pellet or bullet entry paths when game are hunted [1]. These embedded fragments of metallic Pb are a source of dietary Pb exposure for human consumers of wild game. This may pose a significant health risk, especially for subsistence hunting communities that rely on hunter-killed wild game as a major food source [2]–[3]. For example, approximately 7% of Inuit newborns from northern Quebec (Canada) between 1993 and 1996 had cord blood Pb concentrations ≥0.48 µmol/ml (or 10 µg/dL), the intervention level adopted by Canadian authorities for young children [3]. In comparison, only 0.16% of Caucasian newborns from southern Quebec had cord blood Pb above this level. The Pb isotopic ratios also differed between these two populations, with ratios in the northern population being closer to that of Pb ammunition signatures [3], and similar results have been obtained when comparing other Canadian populations [4]. In northern Ontario, 24.6% of naturally exfoliated teeth from children who regularly consumed game meat had elevated dentine Pb (≥10 µg/g). These levels were comparable to those found in children living near smelters or in urban areas [2]. The frequency of consumption of wildfowl killed with Pb shot in northern Ontario has also been correlated with Pb in maternal blood and in cord blood [5]. In Greenland, people who ate hunted seabirds up to 2–3 times/month had blood Pb ∼7.5 µg/dL, while those eating seabirds 1–3 times/week had 10.95 µg/dL. At 4–6 times/week, levels were 11.7 µg/dL, while daily consumers had 16.98 µg/dL [6]. In North Dakota, those that ate wild game and those that did not, had blood Pb of 1.27 µg/dL and 0.84 µg/dL, respectively [7]. However, although these levels were significantly different, both were well below levels detected previously in subsistence hunting communities [5]–[6] and a currently established level of concern (10 µg/dL) [3]. Moreover, the frequency of game meat consumption in North Dakota was not associated with increased blood Pb, and only consumers eating >56.7 g of game meat/meal showed higher blood Pb [7]. Likewise, in a Swiss study, blood Pb in hunters (2–17.1 µg/dL) was not associated with the frequency of game consumption [8].

In Spain, small game (mainly partridge, rabbit and quail) are frequently cooked with vinegar (‘escabeche’) and this has been noted to cause increased transfer of Pb from gunshot residues left in meat (when compared with meat cooked without vinegar) [9]. In a similar study, comparable cooking methods (acidic or non-acidic) were also used on various gamebirds, but differences in the total Pb concentration in cooked meat were not found. In this case, the presence and number of small metallic Pb fragments left by the ammunition used was the key defining factor [10]. Although it is known that metallic Pb is less bioaccessible to mammals than are certain highly soluble Pb salts or rapidly disassociating complexes [11]–[12], cooking can promote the transfer of solubilised Pb (from the metallic Pb ammunition surface) to the meat [10]. For example, whole Pb shot manually embedded into otherwise ammunition free meat can promote elevated Pb concentrations throughout subsequently cooked meat (embedded pellets were removed before analysis), in proportion to the number of shot embedded [9]. Such experiments clearly demonstrate that metallic Pb particles in game meat can undergo dissolution, and that the soluble salts generated can readily leach into otherwise lead free meat. In this form, the Pb may also be more bioaccessible when eaten and may therefore pose a greater risk than might the less soluble (at the pH prevailing in the human intestine) metallic Pb particles alone.

The *in vivo* digestive uptake of Pb from Pb ammunition fragments embedded in game meat has now been assessed using an animal model [13]. However, little work has been published regarding the bioaccessibility (the total soluble fraction of a pollutant) and bioavailability (the proportion of that pollutant actually absorbed into the circulatory system by the organism) of Pb ammunition residues in game meat. In soil, several *in vitro* gastrointestinal simulations and *in vivo* models have been developed and used to study Pb bioaccessibility [14]–[18], but such tests have not yet been applied to the exposure scenario described here. In the present work, we present an *in vitro* simulation to compare Pb ammunition residue bioacccessibility in game meat cooked with different recipes. The aim is to provide information that may help food quality regulators, that may inform consumers of game meat as to how to reduce their exposure to Pb (via changes in cooking practices) and that may be employed to adjust existing theoretical models regarding Pb uptake and subsequent risk in humans.

## Materials and Methods

### Sample collection and X-ray

**Figure 1:**
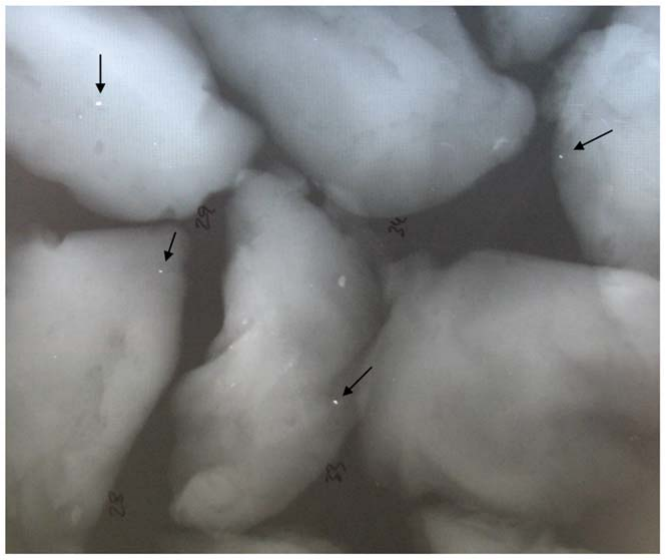
X-ray of breast muscle tissues from red-legged patridges containing small fragments of Pb left by the Pb shot pellets that were used as ammunition to hunt the birds. Fragments are generated as whole shot passes through tissue and fragments on impact.

Sixty-four red-legged partridges (*Alectoris rufa*) were collected during a driven hunt in Albacete (central Spain; for further details see [19]). As partridges were not killed specifically for this study, the approval of the Ethics Committee of the University of Castilla-La Mancha was not required. Birds were X-rayed in order to determine the presence of whole Pb shot pellets in the entire partridge body. The pectoral muscles (left and right) were then removed and X-rayed again, separately, to detect both whole Pb shot and any visible small fragments (<1 mm) within the meat (Fig. 1). The number of Pb shot were recorded, as were the number of Pb fragments. The latter were scored as zero visible, low numbers (1–2 fragments) or high numbers (≥3).

### Cooking treatments

The pectoral muscles (n = 128) were assigned to 4 balanced groups according to the number of whole Pb shot and shot fragments recorded (with each group having a suite of samples containing the full range of residues). The first three groups (32 breasts each) were then cooked with three different traditional Spanish recipes used for small game meat: (1) ‘escabeche’ where vinegar is added before cooking (as used in [9]; final pH after cooking was 4.5–4.7), (2) ‘escabeche’ where vinegar was added just after cooking (final pH was 4.2–4.5), and (3) ‘a la Toledana’ which uses white wine (pH 5–5.2). The 4^th^ test group was not cooked/simply left raw. After cooking, whole Pb shot pellets were removed from each breast, as is normal when eating game, since the consumer will commonly detect and remove whole shot while masticating. The meat was then finely minced (simulating mastication) and processed to simulate human digestion following a previously described *in vitro* procedure [16], with some modifications based on other models [15], [17], [20].

### *In vitro* gastrointestinal simulation

Meat (10 g wet weight; raw or cooked) was mixed with 30 ml of simulated gastric juice (pre-heated to 37°C) composed of 0.15 M NaCl and 1% porcine pepsin, adjusted to pH 2 with HCl (37%). All mixtures of meat and gastric juice were then adjusted to pH 3 with HCl to minimise effects caused by slight differences in solution pH during this extraction step. The homogenate tube (50 ml polypropylene centrifuge tube) head-space was then purged with argon to minimise the amount of oxygen present. Tubes were placed on an orbital shaker for 2.5 h at 37°C. Next, 1 ml of intestinal juice was added. This was composed of porcine bile extract (0.14 g/ml; Sigma) and pancreatin (0.014 g/ml; Sigma). The final concentration of these reagents in each tube was 3.5 mg/ml and 0.35 mg/ml respectively [16]. The pH was then adjusted to 6.5 with a saturated NaHCO_3_ solution. The homogenate, after argon purging, was again mixed for 2 h at 37°C. An aliquot of the final homogenate (4 g) was then removed by pipette and centrifuged at 14,000 *g*. The supernatant liquid and the solid meat pellet were then acid-digested separately and analysed for Pb.

### Lead analysis and quality control

The meat pellet was pre-digested with 1 ml of HNO_3_ (69% Analytical Grade, Panreac) at room temperature overnight. This solution was then transferred to a quartz digest tube and heated overnight at 70°C. Thereafter, 1 ml of H_2_O_2_ (30% v/v Suprapur, Merck) was added and the tube heated to 110°C for 6 h. These digest solutions were diluted to a final volume of 15 ml with Milli-Q. The supernatant liquid was also digested in 15 ml polypropylene tubes by addition of 1 ml of HNO_3_. Tubes were left at room temperature overnight and then heated for 1 h at 90°C in a water bath. Thereafter, 1 ml of H_2_O_2_ was added and the tubes heated again at 90°C for 2 h. This digest was diluted to a final volume of 10 ml.

Lead analysis was achieved using graphite furnace - atomic absorption spectroscopy (GF-AAS; AAnalyst 800, Perkin Elmer) using 50 µg of NH_4_H_2_PO_4_ and 3 µg of Mg(NO_3_)_2_ as matrix modifiers in each atomization. Calibration standards were prepared by dilution of a commercial certified stock solution containing 1 g/l of Pb (Panreac). Blanks (n = 7), and digested ammunition free raw meat homegenate phases (pellets and supernatants) were also spiked with 1.5 µg of Pb (n = 3) and analysed for Pb using the same methods. The limit of detection (LOD) for Pb calculated using the blank data was 0.007 µg/g for the soluble Pb fraction (supernatant) and 0.038 µg/g for the insoluble Pb fraction (pellet). Samples containing Pb levels below the LOD were assigned a level equal to half the LOD (for statistical purposes). Recovery % (mean±SE) for the spiked samples was 96.4±5.5%. Reference samples of lobster hepatopancreas (TORT-2, National Research Council, Canada; n = 3) with 0.35±0.13 µg/g Pb, and bovine liver (CRM 185R, Community Bureau of Reference, EU; n = 3) with 0.172±0.009 µg/g Pb were analysed. Lead concentrations obtained were 0.31±0.06 µg/g and 0.169±0.014, respectively.

### Bioaccessibility calculations

Bioaccessible (soluble) and non-bioaccessible Pb (insoluble) in the cooked/raw meat were calculated using the level of Pb measured in the supernatant and the pellet digests, respectively. These values were multiplied by the post-digestion dilution factor (10 or 15 for supernatant and pellet, respectively), then divided by the total homogenate mass analysed (∼4 g - exact weight recorded during experiment), then multiplied by the ratio between the entire homogenate mass at the intestine stage (∼41 g – exact weight recorded) and the entire meat mass in the homogenate (∼10 g – exact weight recorded). The total Pb concentration in the homogenate was calculated as the sum of the supernatant and meat pellet concentrations. Bioaccessibility was calculated as the percentage of bioaccessible (soluble) Pb in the intestinal phase with respect to the total Pb concentration (in those samples with detectable total Pb levels). All concentrations are expressed in µg/g wet weight (w.w.). In gastrointestinal *in vitro* simulations such as this, Pb bioaccessibility in the intestinal phase has previously been suggested to provide a more reliable indication of *in vivo* Pb bioaccessibility (in comparison to measurements based on the gastric phase of such simulations, [14], [18]).

### Risk modelling process

In order to consider the potential risk posed by consuming ammunition fragments embedded in cooked game meat, the results presented here have also been analysed using the US EPA's Integrated Exposure Uptake Biokinetic model for Pb (IEUBK win32 Lead Model Version 1.1 Build 11) [21]–[22]. The IEUBK model is based on several assumptions, including the bioavailability of Pb from different sources (i.e., soil, dust, diet, water). The model also permits the inclusion of alternative food items, such as game meat, and allows dietary Pb bioavailability to be modified. In order to utilise the model, we first had to transform our observed bioaccessibility data into bioavailability values as follows. *In vitro* and *in vivo* Pb bioaccessibility or bioavailability (for contaminated soil) are sometimes expressed in relative terms, i.e., with respect to values obtained for a reference material/dietary source such as Pb acetate [14], [16], [18]. The solubility of Pb acetate at an intestinal pH of 6.5 has been previously calculated as 14.3% [18]. We can therefore estimate here, a relative intestinal bioaccessibility for Pb from ammunition in our game meat by dividing our observed bioaccesibility values by 0.143. The bioavailability of Pb in diet (i.e., the proportion of the bioaccessible fraction that is actually absorbed into the circulatory system) in adults is normally noted to be around 10–15% [23], however, in children it is considered to be higher, i.e., approximately 50% [22], [23]. Therefore, we can estimate (after multiplying by this (0.5) correction factor; [15]) the absolute Pb ammunition residue bioavailability in children. In the IEUBK model results presented, we therefore used the total mean Pb level in our game meat (2.55 µg/g), two calculated absolute Pb bioavailability figures (for the wine and vinegar recipe data) and varied the contribution of total meat intake derived from our game meat from 0 to 100% (see Results for further details). Model calculations provide the mean blood Pb level that would be expected to be attained, and the % of children (with age 0–7 years) that would be expected to have >10 µg/dL blood Pb. These calculations were also performed assuming relatively low Pb exposure via other sources (i.e., from soil and dust: 10 µg/g; air: 0.01 µg/m^3^; water: 1 µg/L).

### Statistical analyses

Total Pb concentrations and the bioaccessible fraction (soluble Pb at the end of the intestine phase simulation) were log-transformed to approach a normal distribution. Normality and homogeneity of variance were not attained for many of the variables for each of the experimental groups. Therefore, non-parametric Kruskal-Wallis and Mann-Whitney tests were used to detect differences in results between various cooking treatments, and to study the effect of the presence of Pb ammunition (whole shot pellets and/or fragments) in the meat. Differences between cooking treatments were studied separately in samples with and without Pb ammunition. Bioaccessibility differences among cooking treatments were also tested using Kurskal-Wallis tests. The relationships between total Pb and bioaccessible Pb (both log-transformed) were examined using linear regressions to detect slope differences among cooking treatments. Differences among slopes were studied using analyses of covariance, noting where significant interactions occurred between total Pb (as an independent variable) and cooking treatment (as a factor). Statistical analyses were performed with SPSS 17.0 and the p value for significance was set at 0.05.

## Results

For the whole partridge carcasses, 87.5% contained X-ray visible Pb shot pellets, ranging in number from 1 to 22. In the 56 partridges containing pellets, the mean number of pellets was (arithmetic mean±SE) 4.20±0.33 and the median number was 4. In X-rayed pectoral muscles, 10.2% contained whole Pb shot pellets (8.6% having one pellet, 1.6% having two) and 60.9% had small Pb fragments. The total mean Pb concentration in all samples (n = 128) was 2.55±0.75 µg/g, and higher in pectoral muscles with small Pb fragments (n = 78, 3.41±1.17 µg/g) than in those without (n = 50, 1.22±0.57 µg/g; Z = 2.795, p = 0.005). However, a difference was not observed between samples with or without whole Pb shot pellets (p = 0.27) or with a high (≥3) or low (1–2) number of fragments (p = 0.26). Samples with evidence by X-ray of any embedded ammunition (presence of pellets and/or small Pb fragments) showed higher total Pb levels than samples without any ammunition (Z = 2.727, p = 0.006; Table 1). In samples containing any embedded Pb ammunition, total Pb did not differ among cooking treatments (p = 0.71; Table 1).

**Table 1:** Total and bioaccessible concentrations of Pb (in µg/g wet weight) in red-legged partridge breast meat cooked using different methods and then subjected to an *in vitro* gastrointestinal simulation.

Recipe	Presence of Pb ammunition by X-Ray*					
	No			Yes		
	n	Mean±SE	Range	n	Mean±SE	Range
	**Total Pb**					
Raw	14	1.83±0.79	(ND – 10.33)	18	5.78±4.50	(ND – 81.14)
Cold vinegar	15	1.97±1.77	(ND – 26.58)	17	4.90±2.24	(ND – 34.85)
Hot vinegar	7	0.03±0.004	(ND – 0.05)	25	2.04±0.67	(ND – 13.64)
Wine	11	0.43±0.24	(ND – 2.14)	21	1.34±0.44	(ND – 6.76)
All	47	1.28±0.61^b^	(ND – 26.58)	81	3.29±1.12^a^	(ND – 81.14)
	**Bioaccessible Pb**					
Raw	14	0.023±0.014	(ND – 0.180)	18	0.010±0.007^C^	(ND – 0.127)
Cold vinegar	15	0.075±0.065	(ND – 0.985)	17	0.259±0.095^A^	(ND – 1.393)
Hot vinegar	7	0.004±0.000	(ND – 0.004)	25	0.164±0.052^AB^	(ND – 0.835)
Wine	11	0.019±0.014	(ND – 0.162)	21	0.044±0.020^BC^	(ND – 0.326)
All	47	0.036±0.021^b^	(ND – 0.985)	81	0.119±0.028^a^	(ND – 1.393)

Values given according to the presence of Pb ammunition (whole shot and/or fragments)*.

*Whole Pb shot pellets were removed from the meat before samples were homogenised and subjected to the *in vitro* simulation.

Means sharing an uppercase letter do not differ among cooking treatments. Lower case letters show where there was a significant difference between the total sample mean for meat with or without identified Pb ammunition.

Mean bioaccessible Pb in all samples was 0.088±0.019 µg/g. Differences in bioaccesibility were found between cooking treatments in samples containing Pb ammunition (χ^2^_3_ = 9.96, p = 0.019). As shown in Table 1, the bioaccessible Pb fraction was higher in meat cooked with vinegar, independent of when the vinegar was added to the meat (before or just after cooking). The bioaccessible fraction was also lowest in raw meat, and intermediate when using the recipe with wine (Table 1). Differences in bioaccessible Pb among treatments were not significant for samples without Pb ammunition (p = 0.74; Table 1).

**Figure 2:**
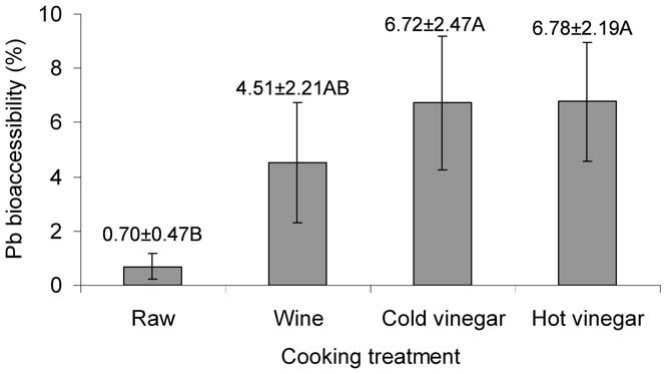
Mean±SE percentage Pb bioaccessibility in red-legged partridge breast meat as a function of the cooking treatment used on the meat before it was subjected to an *in vitro* human digestion simulation. Values shown here include all meat samples, with and without evidence of Pb ammunition by X-ray. Bioavailability values have been calculated for all samples, not just those containing Pb ammunition residues. Means sharing the same uppercase letter were not significantly different (Kruskal-Wallis test).

**Figure 3:**
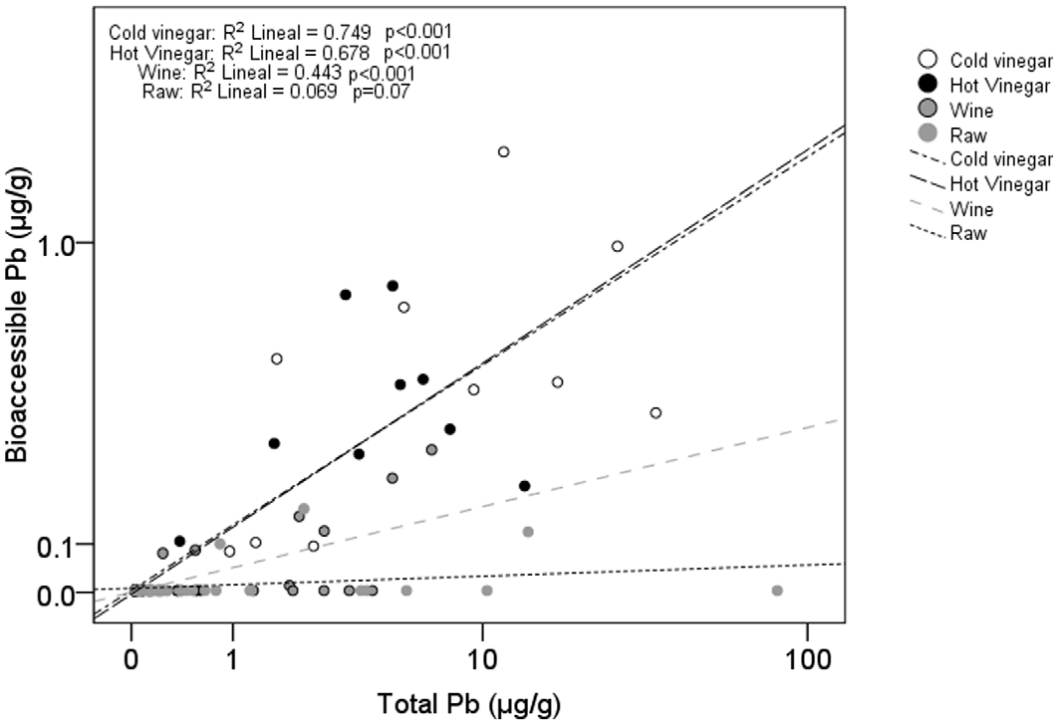
Total versus bioaccessible Pb (µg/g) in red-legged partridge breast meat cooked using various methods before being subjected to an *in vitro* human digestion simulation.

In terms of percentage bioaccessibility, when including all samples (with and without Pb ammunition), the highest percentages were found in samples cooked with vinegar, then in those cooked with wine, and then finally in the raw meat (χ^2^_3_ = 16.96, p = 0.001; Fig. 2). Bioaccesiblity values for meat cooked with wine and vinegar (a mean of the two vinegar recipes) were 4.51% and 6.75%, respectively, whereas raw meat had a far lower value of 0.7%. These differences can also be seen in the regressions between the bioaccessible and total Pb levels (Fig. 3). The line slopes were higher and very similar for samples cooked with vinegar, followed by those cooked with wine (although this was not significantly different to those involving vinegar), and finally in the raw meat (where the slope was significantly different from the other three treatments; F_3, 120_ = 11.55, p<0.001).

The relative intestinal bioaccessibility for Pb from ammunition in our game meat, with respect to Pb acetate (see Methods), was 31.5% (4.51%/0.143) for the wine recipe used, and 47.2% (6.75%/0.143) for the vinegar recipes. Therefore, we can estimate that the absolute Pb ammunition residue bioavailability in children here would be 15.7% (31.5%×0.5; for the wine recipe) and 23.6% (47.2%×0.5; for the vinegar).

**Figure 4:**
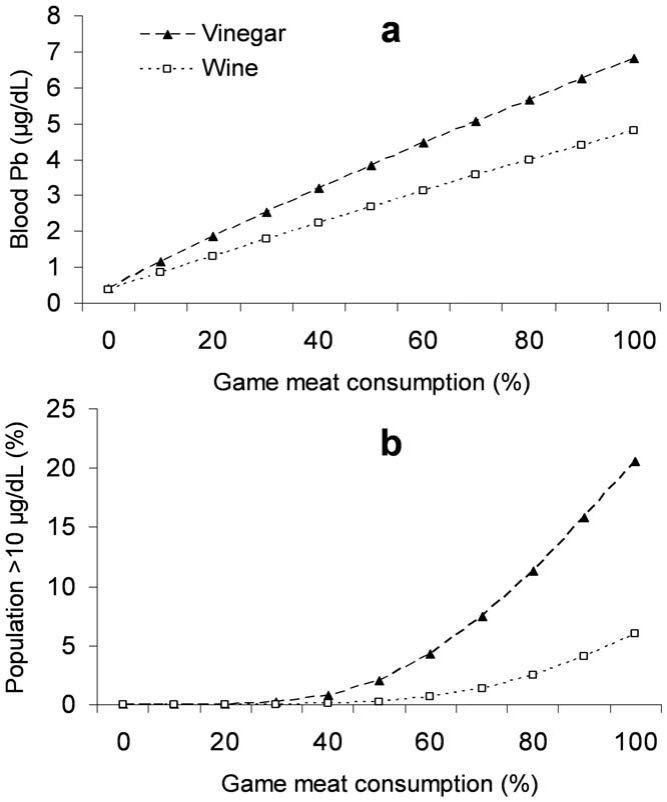
Results obtained when using the US EPAs Integrated Exposure Uptake Biokinetic model for Pb[21]–[22] and the Pb levels in game meat and Pb bioavailability derived from the present study. Upper graph denotes the blood Pb level which would result from eating red-legged partridge breast meat (as 0–100% of total meat intake in diet) when cooked using two traditional methods (‘escabeche’ with vinegar, and ‘a la Toledana’ with wine). Lead levels are plotted for 0–7 year old children. Lower graph denotes what percentage of these children would have blood Pb>10 µg/dL assuming the same consumption rates. The bioavailability of Pb in meat cooked in vinegar was 23.6% while it was 15.7% for the wine based cooking method.

In the IEUBK model, we therefore used a total mean Pb level in game meat of 2.55 µg/g, two absolute Pb bioavailability figures (15.7 and 23.6%) and varied the contribution of total meat intake derived from our game meat from 0 to 100%. Model results provide the mean blood Pb level that would be attained (Fig. 4a), and the % of children (age 0–7 years) that would be expected to have >10 µg/dL blood Pb (Fig. 4b). These calculations also assume relatively low Pb exposure via other sources (i.e., from soil and dust: 10 µg/g; air: 0.01 µg/m^3^; water: 1 µg/L) and assume the same dietary bioavailability (15.7 or 23.6%) for all dietary items. Fig. 4b clearly shows that there is a dramatic increase in the proportion of children that would be expected to have blood Pb >10 µg/dL even when only quite small increases in Pb bioavailability occur (from 15.7 to 23.6%). This effect is obviously more marked where game meat consumption levels are high.

## Discussion

In 54.7% of all samples, total Pb levels were above the maximum residue level (MRL), which is 0.1 µg/g w.w., established for farm reared meat (chicken, beef, lamb, etc) in EU countries [24]. The percentage exceeding this level was higher in samples containing Pb ammunition (pellets and/or fragments, 61.7%) than in those that did not (42.6%). If we were to consider a 200 g serving of meat to be a normal size portion of small game [25], a provisional tolerable weekly intake (PTWI) of Pb of 25 µg/kg body weight [26] would be exceeded by just one meal in 7 to 8.6% of cases (assuming an adult weighs 50–70 kg, the PTWI would be 1250–1750 µg Pb/week). The existing/current intake of Pb via the human diet (excluding game consumption) has been studied in several regions in Spain. Results indicate that between 28.4 and 574 µg/day [27]–[30] may be consumed, with a mean of 48 µg/day [31]. By eating 200 g of the red-legged partridge meat analysed here (with an overall mean Pb level of 2.55 µg/g), 510 µg of Pb would be ingested by this dietary source alone. This is 10.6 times the estimated mean daily intake noted above (48 µg/day). If we consider an estimated existing weekly intake in Spain is 336 µg, just 3 meals/week including these partridges would be enough to exceed a PTWI of 1750 µg (i.e., 1530 µg from the game meat plus 336 µg from the rest of the diet). In Spain, game meat is frequently consumed by the families of hunters, and this consumption is not simply restricted to the hunting season (August to February). Hunted prey is commonly stored frozen and consumed throughout the year. Moreover, the hunting season is often essentially extended for the majority of the year in many regions, since special permits can be given to hunters to control wild boar and rabbit populations.

As Pb shot passes through the flesh of a hunted animal, its pathway becomes contaminated with small Pb fragments as the shot disintegrates. Here, the presence of small fragments was a clearer determinant of the total Pb concentration in the meat than was the presence of whole shot (which we removed after cooking but before undertaking the *in vitro* simulation). The practical importance of this finding is that detection/removal of whole Pb shot using metal detection or X-rays, although important, would not ultimately prevent game meat contamination. In reality, the complete removal of all small Pb ammunition fragments in small (or large) game through meat processing practices is almost certainly impossible. In Canada, waterfowl killed with Pb shot also had very high Pb (3910 µg/g d.w.) in breast meat [1]. This contamination remained within the meat even after all visible Pb shot were removed. Subsequent radiography showed that numerous smaller (<1 mm diameter) Pb fragments were present in pectoral muscle tissues from these birds, which explained the Pb contamination reported. These authors also found that the mean Pb level in meat from pooled gamebirds was 12 µg/g, i.e., ∼5 times higher than the total mean Pb concentration described here. In Greenland, Pb in meat from hunter-killed seabirds boiled in salted water (a traditional recipe called ‘suaasat’) was 0.22 µg/g w.w. This was also 10 times higher than in meat from birds not killed with Pb shot [25]. Further work showed that mean Pb was 6.1 and 0.73 µg/g in breast meat from thick-billed murres (*Uria lomvia*) and common eiders (*Somateria mollissima*), respectively, when killed with Pb ammunition [32]. In the UK, red-legged partridge and common pheasant (*Phasianus colchicus*) shot with Pb had levels >0.1 µg/g in 56.1% and 46.6% of raw meat, respectively. Mean Pb in cooked meat from these species in the UK was also 1.12 and 0.98 µg/g, respectively [10].

By transforming our observed bioaccessibility data into relative bioavailability data, we have been able to evaluate the potential risk posed by consuming ammunition fragments embedded in cooked game meat using the US EPA's Integrated Exposure Uptake Biokinetic (IEUBK) model [21]–[22]. Previously, the IEUBK model for Pb has also been used to consider the effect on children of consuming mourning dove (*Zenaida macroura*) meat contaminated with Pb [33]. This study showed that for 5 year old children, who consumed 120 g of dove meat/day with a mean Pb level of 0.12 µg/g, their geometric mean blood Pb may rise to 4.4 µg/dL. This estimated level would be 4 µg/dL in children who did not eat the dove meat, and 6.6 µg/dL in those that ate dove meat with 1.6 µg/g Pb (the maximum detected). This study assumed a default value for absolute dietary Pb bioavailability of 50%. We have calculated here that the mean absolute Pb bioavailability in game meat cooked with wine or vinegar was actually likely to be somewhat lower than this 50% assumption (i.e., 15.7% and 23.6%, respectively), and this fact would therefore affect any model estimations generated. Fig. 4b shows that an important increase in the proportion of children that would be expected to have blood Pb>10 µg/dL occurs when only quite small increases in Pb bioavailability occur (from the 15.7% to 23.6% level). In the IEUBK model, if we use a total mean Pb level in game meat of 2.55 µg/g and assume relatively low Pb exposure via other sources, the mean blood Pb level in children (aged 0 to 7 years) would be 4.8 µg/dL when eating our game meat cooked with wine (if this were the only meat in their diet). This concentration would increase to 6.8 µg/dL when ingesting meat cooked with vinegar based recipes. Therefore, our results indicate that by using wine instead of vinegar in cooking such game, the percentage of children with >10 µg/dl of Pb in blood could be reduced from 2.08% to 0.26% (when game meat represents 50% of meat in the diet; Fig. 4b). To put these results in context, levels above 10 µg/dL in blood have had clearly documented significant negative effects on cognitive ability in children [34]–[35]. In fact, the average IQ reduction per µg/dL increase in blood Pb over the 1 to 10 µg/dL range has been estimated to be 0.69 IQ points [35]–[36]. Game meat cooked with either wine or vinegar, which had Pb levels in accordance with existing EU regulations (<0.1 µg/g) would result in mean blood Pb values in children of <1.2 µg/dL, and the percentage with >10 µg/dL would be <0.001%, even when 100% of meat consumed was from such game. This clearly indicates that the best way to reduce the risk of elevated Pb exposure in children via this pathway, is by ensuring that only non-toxic, Pb free ammunition is used across the EU (and beyond).

The blood Pb levels and the population percentages exceeding 10 µg/dL shown in Fig. 4 are valuable in the sense that they help compare the effect of different cooking methods. Also, the estimated values show some agreement with existing data regarding *in vivo* and epidemiological studies. For example, blood Pb in pigs fed venison meat with embedded Pb ammunition for two days increased to 2.29 µg/dL (from a control level of 0.63 µg/dL; [13]). Therein, meat was also only cooked using a microwave oven, hence, it seems entirely feasible that higher blood Pb levels might be attained when more acidic cooking recipes are used (which aids Pb release into the meat). Moreover, existing data regarding blood Pb in children from subsistence communities where hunter-killed wild game is a major food source have been demonstrated to be similar to those estimated here. For example, geometric mean Pb in cord blood was 4 µg/dL (in northern Quebec), and 7% of newborns had circulating levels >10 µg/dL [3]. Likewise, in Ontario, mean blood Pb was 2.3 µg/dL in mothers, 2.1 µg/dL in umbilical cord blood and 1.7 µg/dL in 4 month old infant blood [5]. Once again though, the consumption by children of game meat cooked with acidic recipes would probably cause even higher blood Pb levels.

In future, further research will be needed to establish better/more comprehensive bioavailability and bioaccessibility data for this particular food contamination source. Also, by characterising the Pb compounds that are formed in game meat containing Pb ammunition residues during cooking, a better understanding of the solubility of such Pb sources in the gastrointestinal tract could be gained [12], [34]. Here, by improving our understanding of the bioaccessibility generated when using different cooking methods, useful recommendations can be made for human consumers of hunted game. Our data quite clearly indicate that when using recipes involving wine rather than vinegar, exposure to more soluble forms of Pb in the game meat can be reduced. The bioaccesibility of Pb from ammunition in the recipes used here was actually independent of the moment in time when the vinegar was added to the meat (i.e., before or after cooking). This was somewhat unexpected, since the time spent in contact with the acidic vinegar did not have a significant effect on Pb transfer to the game meat (as ahown in a previous study by our group [9]). Previously, we concluded that heat and vinegar together were primarily responsible for driving Pb transfer to the meat. Here, where vinegar was added after cooking, it was added while the meat was hot, hence transfer to the meat may have occurred during the short period while the meat cooled. Likewise, the presence of acetic acid on/within the meat may have promoted the formation of bioaccessible Pb salts during the gastric simulation (even though the pH was equal in all experiments). Ultimately, cooking recipes that do not involve acidic ingredients such as wine/vinegar should also be tested. In previous work, significant reductions were observed in the transfer of Pb from Pb shot to game meat when recipes involved water rather than vinegar [9].

The only existing MRLs for meat in the EU currently refer solely to livestock meat (chicken, lamb, beef, etc), in part because game meat is normally eaten far less frequently by the general population (although it may be very important for certain sub-populations). This legislative omission should be reconsidered in future regulations regarding MRLs for human food [9]–[10], [37]. Of particular concern in terms of Pb ammunition, is the fact that game meat consumption can actually be quite high amongst the families of hunters, or, in certain rural or geographic areas within Europe. These consumers should be aware of the potential risks involved in consuming game that may be Pb contaminated. Moreover, as a definitive/optimum solution, if existing non-toxic hunting ammunition alternatives were to effectively replace the use of Pb based products, the risk of Pb exposure for game meat consumers would inevitably be greatly reduced.
